# Pelvic floor muscle training in radical prostatectomy: a randomized controlled trial of the impacts on pelvic floor muscle function and urinary incontinence

**DOI:** 10.1186/s12894-019-0546-5

**Published:** 2019-11-15

**Authors:** Joanne E. Milios, Timothy R. Ackland, Daniel J. Green

**Affiliations:** 1 School of Human Sciences (Exercise and Sport Science), The University of Western Australia, Crawley, Western Australia 6009 Australia; 2 Faculty of Science, School of Human Services (Sport Science, Exercise and Health), University of Western Australia, Parkway Rd, Crawley, Western Australia 6009 Australia

**Keywords:** Pelvic floor muscle, Prostatectomy, Men’s health, Urinary incontinence, Quality of life

## Abstract

**Background:**

Pelvic floor muscle training (PFM) training for post-prostatectomy incontinence (PPI) is an important rehabilitative approach, but the evidence base is still evolving. We developed a novel PFM training program focussed on activating fast and slow twitch muscle fibres. We hypothesized that this training, which commenced pre-operatively, would improve PFM function and reduce PPI, when compared to a control group.

**Methods:**

This randomized trial allocated 97 men (63 ± 7y, BMI = 25.4, Gleason 7) undergoing radical prostatectomy (RP) to either a control group (*n* = 47) performing low-volume rehabilitation, or an intervention group (*n* = 50). Both interventions commenced 5 weeks prior to surgery and continued for 12 weeks post-RP. Participants were assessed pre-operatively and at 2, 6 and 12 weeks post-RP using 24 h pad weights, International Prostate Symptom Score (IPSS), Expanded Prostate Cancer Index Composite for Clinical Practice (EPIC-CP) and real time ultrasound (RTUS) measurements of PFM function.

**Results:**

Following RP, participants in the control group demonstrated a slower return to continence and experienced significantly more leakage (*p* < 0.05), measured by 24 h pad weight, compared to the intervention group, suggesting an impact of the prehabilitation protocol. PFM function measures were enhanced following RP in the intervention group. Secondary measures (IPSS, EPIC-CP and RTUS PFM function tests) demonstrated improvement across all time points, with the intervention group displaying consistently lower “bothersome” scores.

**Conclusions:**

A pelvic floor muscle exercise program commenced prior to prostate surgery enhanced post-surgical measures of pelvic floor muscle function, reduced PPI and improved QoL outcomes related to incontinence.

**Trial registration:**

The trial was registered in the Australia New Zealand Clinical Trials Registry and allocated as ACTRN12617001400358. The trial was registered on 4/10/2017 and this was a retrospective registration.

## Background

Prostate cancer (PCa) is the most commonly diagnosed cancer in men [1]. Radical prostatectomy (RP) is highly effective, with 97% of patients surviving at least 5 years following surgery [2]. However, side effects can be severe and distressing [3, 4]. Stress urinary incontinence (SUI) is the most common presentation following RP and is provoked by activities such as sneezing, coughing, bending, lifting, positional change and exercise [5]. The current gold standard approach to the assessment of UI (urinary incontinence) involves collection of 24 h pad weight scores (‘mild’ < 100 g, ‘moderate’ 100–400 g, ‘severe’ > 400 g) [6]. Anxiety and post-traumatic stress are associated with the combination of PCa diagnosis, the need for surgery and resultant UI [7], with depression occurring four times more often in men with PCa than their healthy counterparts [8].

Randomized controlled trials have investigated the impact of pelvic floor muscle (PFM) training on post prostatectomy UI, providing conflicting evidence. Whilst some support the benefits of exercise [9] other studies (summarized in the recent Cochrane Review [10]) do not recommend PFM training as first line rehabilitation, suggesting that UI symptoms improve over time, irrespective of management. The disparity in these findings likely stems from variability in the type of assessment performed, instructions given to subjects, and mode of delivery of PFM training [11]. Protocols that include pre-operative PFM training that continues post-surgery, and that specifically target fast- and slow-twitch muscle training performed in the clinically relevant standing posture, have not been previously been undertaken. Our primary focus was to lessen the burden of post-prostatectomy UI, since early return to continence has been widely acknowledged as a major quality of life (QoL) outcome [3]. We hypothesized that a significant difference in UI would be observed 2, 6 and 12 weeks post-surgery, when RP patients were randomized to either a PFM training intervention, or a control (‘usual care’) group.

## Methods

Participants were enlisted from a cohort referred sequentially by their Urologist for pre-prostatectomy PFM training to a single physiotherapy clinic, in line with standard procedure; no patient had surgery delayed as a result of enrolment in this study. We used a minimization approach to randomization, with each participant randomly allocated to one of two groups, ‘usual care’ or ‘high intensity’ upon presentation to a high-volume physiotherapy clinic following a diagnosis of PCa. If randomly allocated to usual care, for example, the very next patient was allocated to the high intensity intervention group and this sequence continued until the desired sample was achieved. No a priori consideration was given to other factors such as age, surgical approach, surgeon, aetiology, or BMI. Once consent was provided, enrolment in the study and interventions commenced without delay. Relevant notes were recorded in participant medical files, which were collated over the trial duration, with results subsequently analysed by a blinded, independent statistician.

Patients with pre-existing UI, prior prostate surgery, or a history of receiving radiation or androgen deprivation therapy, were excluded (see Table 1). Both open RP (Control 8, Intervention 5) and Robotic–Assisted Laparoscopic Prostatectomy (RALP) (Control 39, Intervention 45) surgical types, performed by highly experienced surgeons operating at two high volume institutions, were included. All participants were provided with usual care advice regarding bladder training (minimum time between voids 2 hourly where possible), caffeine (maximum 1 serve /day) and alcohol consumption (to be avoided until continent), and behavioural changes relevant to minimizing incontinence issues. In addition, pre-operative physical activity levels were recorded at the initial screening consultation. Type of exercise, frequency, duration and difficulty were recorded and activity levels were rated as ‘low’ (i.e. 40–50% maximum heart rate (MHR)), ‘moderate’ (50–70% MHR), or ‘high’ (70–85% MHR) [13] with the number of sessions per week noted. As part of both intervention arms of the study, all participants were encouraged to walk daily for 30 mins, 5 days per week.

**Table 1:** Inclusion – Exclusion criteria

Inclusion criteria	Exclusion criteria
• Pre-operative radical prostatectomy • Open or robotic-assisted approaches • Age > 18 years • Diagnosed with prostate cancer and referred for pelvic floor muscle training • Fully continent	• Acute illness • Prior urinary incontinence • Current smokers • Diabetes: type 1 or 2 • Alcohol consumption > 21 units/week • Impaired mental status • Prior prostate surgery • Undergoing or had prior radiation therapies • Undergoing or had prior androgen deprivation therapy

### Procedures

#### Pre-surgery PFM training

Five weeks prior to RP surgery, participants were randomized to the intervention or usual care group. Both groups received initial physiotherapy-directed PFM instruction over two sessions of 30 min and were then prescribed a daily PFM training program. This program differed in mode and intensity, depending on randomization. Subjects allocated to the usual care group were instructed and directed to perform three sets of PFM exercises per day, with 10 contractions per set, aiming to hold for a duration of 10 s, with an equal rest time, providing a total of 30 contractions per day. Daily exercise sets were performed once each, in supine, sitting, then standing, in accordance with previously reported interventions [14]. This intervention is in keeping with current clinical practice [15] and it was administered in preference to a ‘no-exercise’ intervention as we considered it unethical to withhold some level of training. Nonetheless, it did not specifically target fast and slow-twitch muscle function and the intensity, number and duration of contractions were modest relative to the intervention group.

Exercise protocols in the intervention group targeted both slow and fast-twitch muscle fibres and participants were required to perform six sets of PFM exercises per day, with each set comprising 10 fast (1 s duration) and 10 slow (10 s duration) contractions with an equal rest time, providing a total of 120 contractions per day. All sets were performed in a standing posture for this group. Participants were blinded to group allocation; in that they were not exercised as a group and were not aware of the existence of the alternative exercise intervention.

During the initial instruction sessions, participants were given written and verbal instructions on correct PFM exercise technique [16] to ensure a full contraction and relaxation cycle was implemented with the cue to *“stop the flow of urine and shorten the penis while continuing to breathe”* [16]. Cues to relax abdominal muscles and avoid breath holding were also communicated, and confirmation of correct technique provided with real time ultrasound (RTUS) used as a biofeedback tool. Participants completed a PFM training diary to record the number, type and position of exercises undertaken on a daily basis. Adherence to the training program in both groups was assessed via individual diary entries during fortnightly physiotherapy appointments.

#### Post-surgery PFM training

Post–operative PFM training recommenced following removal of the catheter. Members of the control group performed three sets per day of the same exercises performed pre-surgery, while members of the intervention group continued their exercise regime with six sets per day of fast and slow-twitch training. Both groups exercised in the postures described above and these protocols were maintained throughout the 12-week assessment period.

#### Outcome measures

Bladder diaries for recording 24 h pad weight (the primary outcome variable) were assessed post-surgery at 2, 6 and 12 weeks as the primary outcome. No participant was incontinent prior to surgery, however, all wore pads after surgery and applied their first pad of the day with an empty bladder. Used pads were placed in a single plastic bag and sealed, then stored in a refrigerator to avoid evaporation. Net weight was calculated by deducting dry pad weight, using a single calibrated digital scale. Any positive net weight, recorded to the nearest 1 g, was deemed indicative of incontinence, with ‘zero’ net weight assessed as no leakage and full continence.

Physical activity, fluid intake and pad weights were recorded in the 24-h bladder diary for each participant. Re-test measures were recorded on the same day of the week to account for variable activity levels, thus avoiding any potential discrepancy between sedentary and more physically active days of the week. Six surgeons, working from two high volume hospitals and all with minimum of 7 years’ experience in performing RP, referred patients into the study. Similar numbers per group were achieved for each surgeon. Nerve sparing was a primary goal of all surgeons who participated in this study as it is usual care practice in this population. Resected nerve matter was stained and weighed by a blinded, independent pathologist within 24 h of surgery, and reported in the Results section. A similar number of unilateral, bilateral and no nerve sparing was achieved in both groups (see Table 2).

**Table 2:** Participant characteristics

Characteristics	Control group (*n* = 47)	Intervention group (*n* = 50)
Age (y)	63.5 ± 6.8	62.2 ± 6.8
BMI	25.4 ± 2.7	25.3 ± 2.7
Pre-surgery training (weeks)	5.1 ± 3.2	5.2 ± 2.8
Gleason score	7	7
Prostate size (g)	49.5 ± 15.5	50.8 ± 18.6
Operation type	8 Open 39 Robotic-assisted	5 Open 45 Robotic-assisted
Nerve sparing procedure	5 Unilateral 39 Bilateral 4 Nil	12 Unilateral 36 Bilateral 2 Nil
Catheter in situ (days)	8.6 ± 3.0	8.1 ± 2.7
Pre-operative Activity Levels^a^		
Low (40–50% MHR)	25	27
Medium (50–70% MHR)	20	20
High (70–85% MHR)	2	3

^a^*MHR* Maximum heart rate

Secondary measures of QoL using the International Prostate Symptom Score (IPSS) [17] and Expanded Prostate Cancer Index Composite for Clinical Practice (EPIC-CP) [18] questionnaires were recorded at baseline (approximately 5 weeks prior to surgery) and again at each post-surgery time point. Participants completed the questionnaires in a quiet private room at the completion of a scheduled appointment within the physiotherapy clinic.

Additional secondary measures of RTUS guided tests of PFM function, including the Rapid Response Test (RRT) and Sustained Endurance Test (SET) [19, 20] were performed by a single operator for each measurement using a commercially available point-of-care ultrasound machine (3.5 MHz sector probe, Mindray DP-30 Ultrasound, 6 U-42000440, China) at each post-surgery time point. Details of the methods used in each of these tests, along with their validity and reliability, are available in recent publications [19, 20].

### Statistics

Outcome data were entered into SPSS (v22.0, SPSS, Chicago, IL) for analysis. A series of two-factor, repeated measures ANOVA (Group x Time) were performed and significance was accepted for all analyses at *p* < 0.05. Where necessary, post-hoc *t*-tests for independent samples were performed to determine the time points at which group scores differed.

The sample size of 101 participants was based on power calculations derived from previous studies utilizing 24-h pad weight as an objective measure of UI. Of 45 RCT studies investigated in the most recent Cochrane review [10], very few recorded 24-h pad weight as an objective measure, resulting in a recommendation for its use in future studies to enhance efficacy. Our study design, which randomized men to intensive pelvic floor muscle (PFM) training versus a usual care intervention, where each group received their intervention both pre- and post-surgery, is novel. For power estimation we selected the paper of Centemero et al. [12] as the closest exemplar. This trial randomized 118 men to a pre-surgery PFM intervention, versus no intervention pre-surgery. Both groups received the same post-surgical PFM training intervention [12]. Pad weight and continence were primary outcome measures, as in the current study. Centemero et al. indicated that 44.1% of subjects in the prehab group were continent at 1 month post–surgery, whereas this number was 20.3% in the no intervention group (ES = 23.8) [12]. This difference was statistically significant (*P* = 0.018). Given a priori assumptions of α = 0.05, a two-tailed test and a sample size of 50 per group (100 total), our study possessed > 70% power to detect a similar effect size observed by Centemero et al. [12].

## Results

Of the 101 participants recruited, 97 (63 ± 7 y, BMI = 25.4, Gleason 7) completed the study, with three participants from the usual care group (*n* = 47) and one participant from the intervention group (*n* = 50) unable to finish due to medical complications, including the need for radiation therapy (*n* = 2) and corrective surgery (*n* = 2). Participants were recruited over a 12-month period from April 2016 until April 2017 with follow up over a subsequent 12 months, until April 2018 when sufficient participant numbers were achieved. There were no appreciable differences in baseline characteristics between the groups, with only a few in each group having open RP rather than robotic-assisted surgery (see Table 2). No participant in either group was incontinent prior to surgery. Prostate size, Gleason score and days of catheterization were also similar between groups, as was rate and type of cavernosal nerve sparing.

**Fig. 1:**
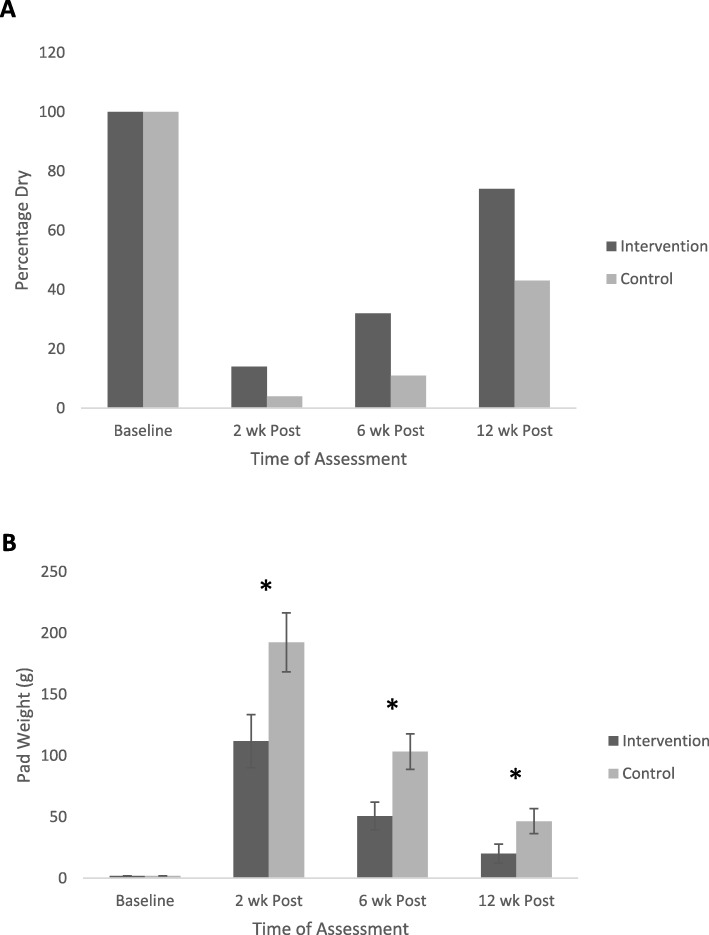
The number of “dry” patients (panel **a**) and changes in 24-h pad weight (panel **b**) for patients following radical prostatectomy within the intervention and control groups at baseline, then at 2, 6 and 12 weeks post-surgery. All participants were fully continent at the pre-operative assessment (baseline). * indicates a significant difference (*p* < 0.05) between groups at the relevant time points

Figure 1a represents the number of dry participants (continence = no pads or 0 g net pad weight), with all participants being continent at the baseline, pre-surgery assessment. At 2, 6 and 12 weeks post-surgery, the percentage of dry participants was greater across all time points in the intervention, compared to control (usual care) group, with the former group demonstrating a faster return to continence. At 2 weeks post-surgery 14% of the intervention group subjects, compared to 4% of the control group, were dry. At 6 weeks, this percentage increased to 32 and 11% respectively, then 74 and 43% by 12 weeks post-surgery.

Figure 1b displays the measured 24 h pad weights for the control and intervention groups pre-surgery and at 2, 6 and 12 weeks post-RP. The ANOVA results showed a significant main effect for Group (*F* = 7.251; *p* = 0.008), Time (*F* = 82.318; *p* < 0.001) and the Group x Time interaction (*F* = 4.204; *p* = 0.016). Post-hoc t-tests demonstrated that group differences occurred at each of the post-surgery time point (*p* < 0.05), however the significant interaction also indicates that rates of improvement differed between groups, favoring enhanced recovery in the intervention group. As we were interested to know whether operation type (RALP vs OPR) influenced the outcome for incontinence, we re-ran the analysis for pad weight using an Analysis of Covariance (ANCOVA) to compare the intervention groups whilst controlling for the effects of operation type. The ANCOVA outputs showed main effects for Time, Group and Group x Time (interaction) remained significant (*p* < 0.05).

**Fig. 2:**
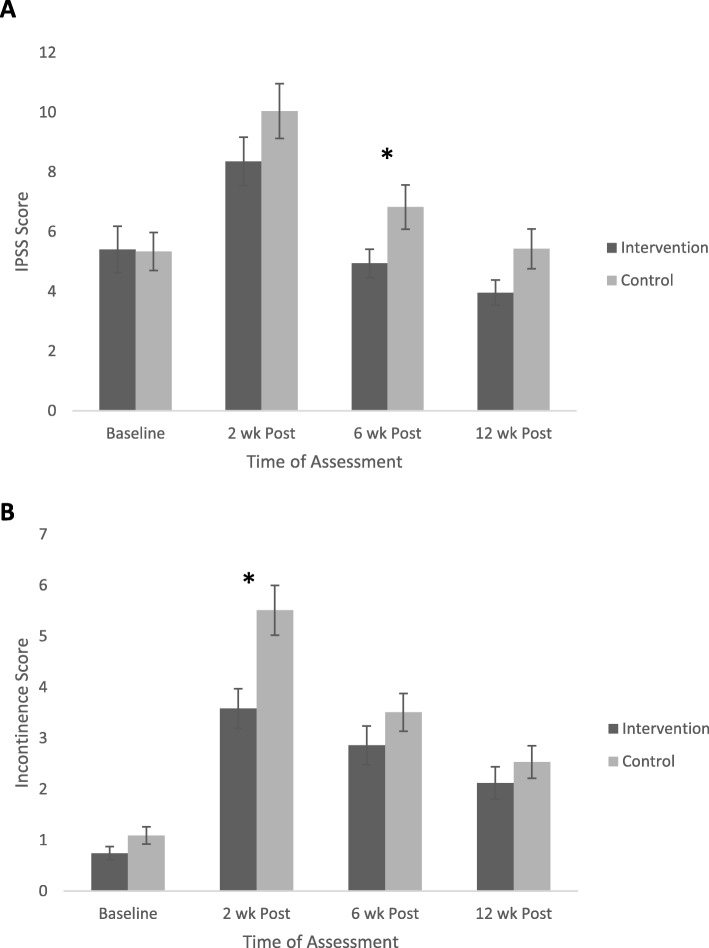
Changes in the International Prostate Symptom Score (panel **a**) and the EPIC-CP (panel **b**) for patients following radical prostatectomy within the intervention and control groups at baseline, then at 2, 6 and 12 weeks post-surgery. The IPSS (maximum score = 12) is as a measure of self-reported urinary symptoms and quality of life, with lower scores indicating better outcomes. The EPIC-CP is a health related quality of life measure for men following treatment for prostate cancer, wherein the urinary continence domain (maximum score = 12) assesses self-reported bother of urinary incontinence symptoms, with lower scores indicating better outcomes. * indicates a significant difference (*p* < 0.05) between groups at the relevant time points

In Fig. 2a, IPSS scores that measure urinary symptoms and QoL perceptions indicated similar levels at baseline, but significant (*p* < 0.05) between-group differences at 6 weeks post-surgery, with the intervention group scores being superior to those for the control group. The ANOVA results showed a significant main effect for Time (*F* = 25.45; *p* < 0.001), but not for Group (*F* = 3.17; *p* = 0.078) or the Group x Time interaction (*F* = 1.261, *p* = 0.288).

Figure 2b describes EPIC-CP urinary incontinence scores (maximum score = 12) for impact on QoL. Pre-surgery EPIC-CP scores were similar, but the intervention group scored significantly better (*p* < 0.05) at 2 weeks post-surgery. There were no other group differences at 6 and 12 weeks post-surgery, although the intervention group retained some advantage. The ANOVA results show a significant main effect for Group (*F* = 5.344; *p* = 0.023) and Time (*F* = 13.844; *p* < 0.001), but not the Group x Time interaction (*F* = 0.486, *p* = 0.487).

**Fig. 3:**
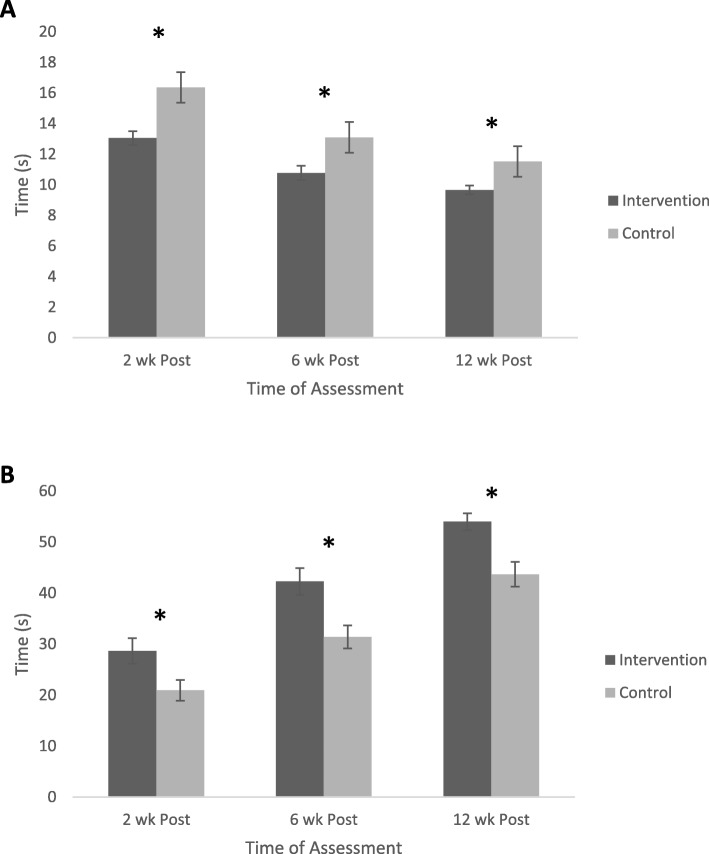
Changes in the Rapid Response Test (RRT – panel **a**) and the Sustained Endurance Test (SET – panel **b**) for patients following radical prostatectomy within the intervention and control groups at 2, 6 and 12 weeks post-surgery. The RRT tests uses real time ultrasound (RTUS) to measure the speed of pelvic floor muscle contractions, with lower scores representing a better outcome. The SET also uses RTUS to measure the endurance of pelvic floor musculature to sustain a contraction over time (maximum score = 60 s), with higher scores representing a better outcome. * indicates a significant difference (*p* < 0.05) between groups at the relevant time points

Results for physiological assessments of pelvic floor muscle function are shown in Fig. 3a and b. Pre-surgery RTUS assessments were not performed so as to avoid any possible training effect for members of the control group participants. However, at all time points post-RP, (Fig. 3a) the intervention group recorded faster (*p* < 0.05) repeated muscle contraction (RRT scores), compared to the control group. The ANOVA results showed significant main effects for Group (*F* = 16.132; *p* < 0.001) and Time (*F* = 69.790; *p* < 0.001), but not the Group x Time interaction (*F* = 2.12; *p* = 0.123). Figure 3b provides results for the sustained pelvic floor muscle endurance test (SET). At all post-surgery time points, the intervention group recorded longer sustained (*p* < 0.05) contraction scores, compared the control group. The ANOVA results show significant main effects for Group (*F* = 12.605; *p* = 0.001) and Time (*F* = 137.671; *p* < 0.001), but not the Group x Time interaction (*F* = 0.679; *p* = 0.508).

## Discussion

The incidence of UI prior to RP surgery is very low, affecting only 1–2% of the male population to age 75 y [21]. Following RP surgery, however, this changes to 69–98% men affected with urinary leakage for some duration [22]. Fear and anxiety of the potential for UI is generally high before surgery, but levels of distress decline as symptoms improve. However, if continence is slow to recover, the long-term negative impact on QoL can remain substantial [23] and with an average 16–51% of men remaining incontinent at 12 months post-surgery, the long term impact on the patient and his partner can be significant [24].

The pre-operative period provides an opportunity to intervene and minimize the impact of UI. With the recommendation of a 6-week period between prostate biopsy and subsequent RP surgery to avoid complications, patients can be referred for pre-operative PFM training. A recent meta-analysis was only able to find five heterogenous papers which had addressed the question of prehabilitation benefit for UI in RP patients and concluded that insufficient data were available to warrant conclusive interpretation [25]. Our results indicate clear outcomes of less leakage, reduced time to return to continence and improved QoL in patients who received more intensive PFM training, utilizing standing postures, compared to the comparator control group protocol. This finding is in keeping with some previous studies which indicate that PFM training of longer duration prior to surgery, or of higher frequency and/or intensity, is more likely to be beneficial [26]. Recent developments in male pelvic floor assessment, utilizing RTUS, ensure that correct muscle activation is executed, with greater focus on anterior PFM, rather than the previously recommended anal sphincter approach. Mastery of PFM control was a novel aspect and focus of our intervention methodology [27, 28], alongside the quantification of pelvic floor muscle adaptation utilizing functional testing with RTUS-informed visual confirmation. Repeated one-on-one physiotherapy training sessions pre-surgery afforded the opportunity to correct any errors in PFM training technique, to progress each patient to sustain 10 s maximal contractions and to allay any participant concerns.

Providing all participants in our study with some level of pre-operative PFM training had the potential to reduce the impact of the group differences we observed and may have been a limitation. However, given the suggestion that PFM training should be recommended as a first line option for the treatment of UI [11], we considered it inappropriate to withhold PFM training altogether in the “control” group. Our intervention group received exercise specifically designed to focus on both fast and slow-twitch fibre training, completed in the upright posture, since this is the posture most often related to the clinical presentation of UI. UI is associated with increases in intra-abdominal pressure during cough, sneeze and sit to stand actions, so it is intuitive for training to be specific to rapid responses to pressure change.

Using RTUS tests that directly visualized and quantified function of the pelvic floor muscles during standardized tests, we were able to demonstrate lower RRT scores across all time points for the intervention versus control group, reflecting faster development of urethral closing pressure. This finding provides physiological and functional data supporting the more clinically orientated outcomes presented above which relate to decreased leakage post-surgery and a quicker return to continence for men who undertook the intervention training protocol. Similarly, training the slow-twitch PFM fibres in the standing posture, as quantified by the SET, resulted in higher scores for the intervention group across post-surgery time points. This was also reflected in reduced leakage and time to continence for the intervention group. No previous PFM exercise research has combined functional imaging-based tests of pelvic floor muscle function with clinical data related to UI to cross-validate findings. Our study is also novel in that we adopted a training protocol targeting both fast and slow-twitch muscle function for men following RP surgery.

Maintaining urethral closure pressure in the standing posture is more demanding than in supine postures, and this is seen clinically with few men leaking in the horizontal position. Upon catheter removal, however, most men will experience significant leakage in transitions from supine to sit, sit to stand, and when bending, lifting and walking. By initiating training in standing postures, the intervention group was able to experience reduced UI in all domains assessed. Matching PFM training to clinical presentations was considered an important issue for our study to address, as opposed to previous studies recommending PFM exercises be performed in sitting or lying postures. Whilst these recommendations may serve patients’ needs when PFMs are weak, the opportunity to train patients pre-surgery may greatly reduce this problem. Furthermore, in physiological studies designed for strength training it is recognized that maximal force generation is achieved by performing three to four sets of 8–12 repetitions per day, which also results in greater hypertrophy of type 2a muscle fibres on biopsy testing [29]. In the context of PFM training in men who experience the complete removal of the prostate, exercise training to achieve some functional change may require more intensive approaches than that previously considered to be ‘usual care’.

Fatigue is one of the major issues of PFM dysfunction and patients routinely complain of worsening incontinence with increased activity, and as the day progresses. To address this issue, participants in the intervention group were prescribed 120 individual maximum PFM contractions per day, compared to only 30 for the control group. Our aim to improve PFM endurance and strength as quickly as possible for the intervention group was achieved by increasing the frequency, number of sets, position and exercise type, versus the usual standard care. Training to increase muscle endurance requires the performance of high numbers of repetitions with decreased recovery time between sets. For example, Kraemer and Ratamess [30] reported greater increases in cross sectional area and strength when participants exercised twice, compared to only once per day. In PFM training studies published to date, wide variability exists in the prescription of exercises for endurance gains, with recommendations to hold PFM contractions ranging from sub-maximal to maximal efforts. In the present study, we chose to prescribe maximal contractions in an effort to increase exercise intensity. Participants recorded their daily PFM exercise regime in a diary which indicated 92% adherence with the PFM training program.

Urinary function and its impact on QoL measures were assessed using the EPIC-CP and IPSS domains, with significant differences found at 2 weeks and 6 weeks, respectively. Men in the intervention group reported less urinary leakage, less irritation, less pad use and less impact on overall QoL. Patients often report a sense of shame, loss of control, fear of bladder accidents and a need for hypervigilance with pad application, fluid consumption and social activity, all of which can affect aspects of daily life. Given the higher levels of depression and anxiety associated with UI post-RP, any measures to reduce these potential outcomes should be encouraged.

There have been many previous studies of PFM training in post prostatectomy patients, delivered both before and after surgery [9, 10, 12]. Many distinct interventions have been assessed and participants have differed in terms of clinical condition and status. Whilst some studies reported positive results and concluded an important role for PFM training, others, including a prominent and large clinical trial and a Cochrane review which included it, suggested that an active intervention did not substantially accelerate the degree of spontaneous recovery. Our findings demonstrated a benefit of the prehabilitation intervention performed at a higher intensity, but we also observed evidence for spontaneous post-op recovery in the control group. There was an increase in the speed of recovery in the more active rehabilitation intervention targeting physiological PFM function of fast and slow-twitch muscle fibres.

There were some limitations in our study, with the first related to sample size. Our power test was based on the paper of Centemero et al. [12] which a priori indicated that a sample size of 50 per group would provide > 70% power to detect a similar effect size for the pad weight and continence outcomes observed in that study. In fact, we observed a larger effect size, with 74% of participants in the intervention group continent at 12 weeks post-surgery, versus 43% in the usual care group (ES = 31), indicating that our study was 87% powered for the groups sizes we achieved (*n* = 50 and 47). Our intervention was more intense that used by Centemero et al. [12] and our index follow-up was longer. We also calculated pad weight in absolute grams and observed a difference between the groups of 20 ± 54 g versus 47 ± 70 g at 12 weeks, indicating that we had 90% power to detect this difference with our sample size [12]. Although the numbers we recruited were adequate for statistical power, we may nonetheless have shown even greater benefit with a larger cohort. In addition, our intervention was for a period of 12 weeks post-operatively, after which, any men who were still incontinent were offered the high intensity PFM protocol. A longer period of intervention may have resulted in further differences between the two groups, with even clearer guidelines for protocol development.

Nerve sparing in our study, was assessed by volume of cavernosal nerve resected with the usual care group having marginally less nerve resected than the intervention group. The relationship between nerve sparing and continence is still emerging, however if this was a critical factor, it may have influenced our results. However, we can conclude in our study, that nerve sparing did not appear to negatively impact the effect of PFM training on continence. Investigations relating to erectile function may display a more significant relationship and this represents another opportunity for research in similar populations.

## Conclusion

In conclusion, an intensive PFM training intervention, applied prior to surgery, improved post-surgical pelvic floor muscle function and decreased UI, compared to a control group. Group differences were also apparent for perceived UI and QoL post-surgery. Continence and PFM functional recovery was observed post-surgery for both groups, with some evidence for more rapid recovery by 12 weeks as a consequence of the more intense intervention. Our findings provide support for further investigation, in larger trials, of the impact of relatively intensive prehabilitative approaches to enhancing PFM function in men with prostate cancer.

## Data Availability

The datasets used and/or analyzed during the current study are available from the corresponding author on reasonable request.

## Abbreviations

BMI: Body Mass Index
EPIC-CP: Expanded Prostate Cancer Index Composite for Clinical Practice
IPSS: International Prostate Symptom Score
PCa: Prostate Cancer
PFM: Pelvic floor muscle
QoL: Quality of life
RALP: Robotic Assisted Laparoscopic Prostatectomy
RP: Radical Prostatectomy
RRT: Rapid Response Test
RTUS: Real Time Ultrasound
SET: Sustained Endurance Test
SUI: Stress Urinary Incontinence
UI: Urinary Incontinence
